# Supplementary material for: Antagonistic effects of chemical mixtures on the oxidative stress response are silenced by heat stress and reversed under dietary restriction
Source: Exposome. Author manuscript; Available in PMC 2025 Oct 16. (PMC12525721; doi:10.1093/exposome/osab005)
Supplement: Supplementary_material [file NIHMS2115504-supplement-Supplementary_material.docx]

**Antagonistic effects of chemical mixtures on the oxidative stress response are silenced by heat stress and reversed under dietary restriction**

Karthik Suresh Arulalan, Javier Huayta, Jonathan W. Stallrich, and Adriana San-Miguel

**SUPPORTING INFORMATION**


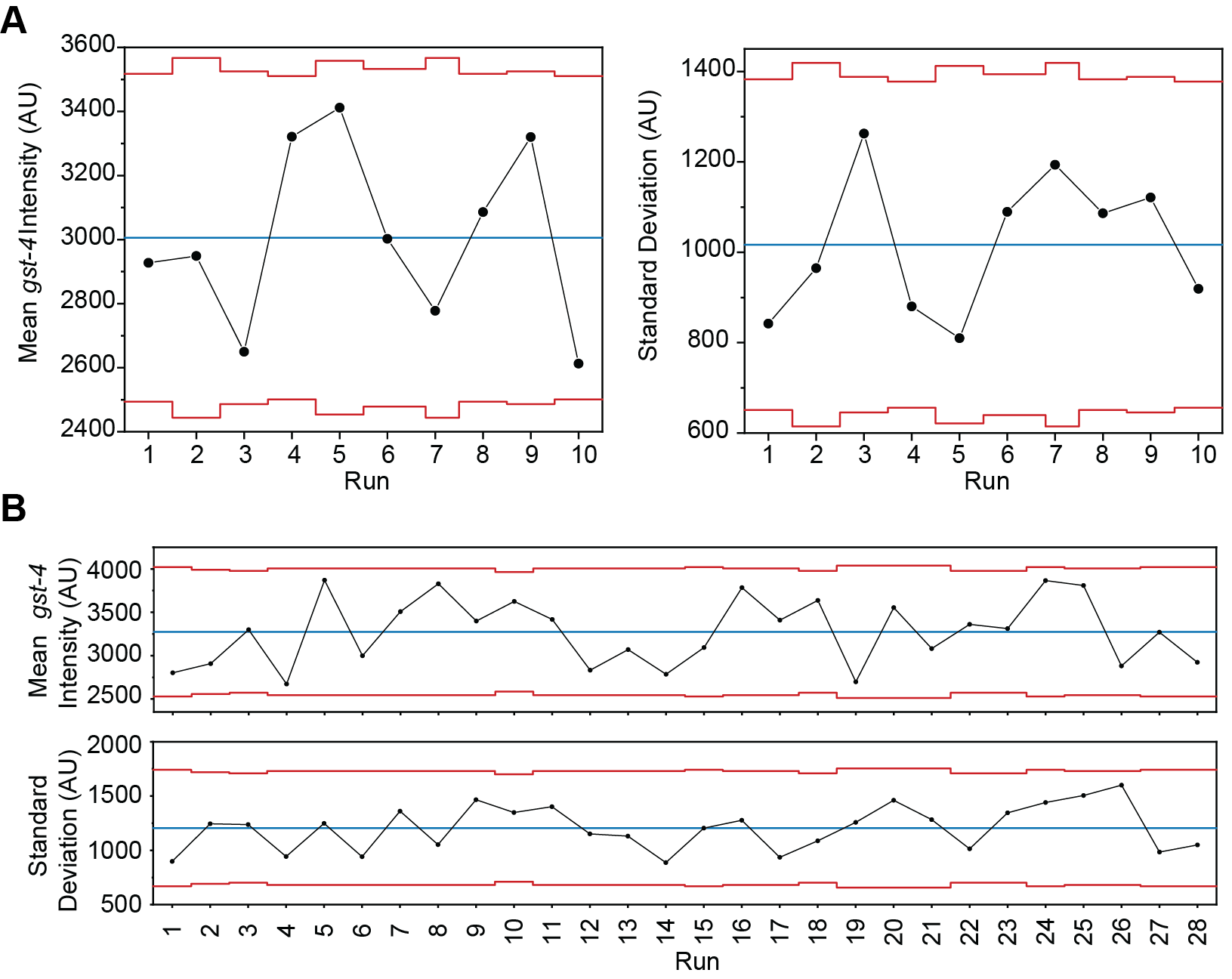


**Figure S1: Xbar and S-charts for populations tested.** A) Xbar and S-chart for 10 populations used for oxidative stress assays. B) Xbar and S-chart for 28 populations used for oxidative stress assays.


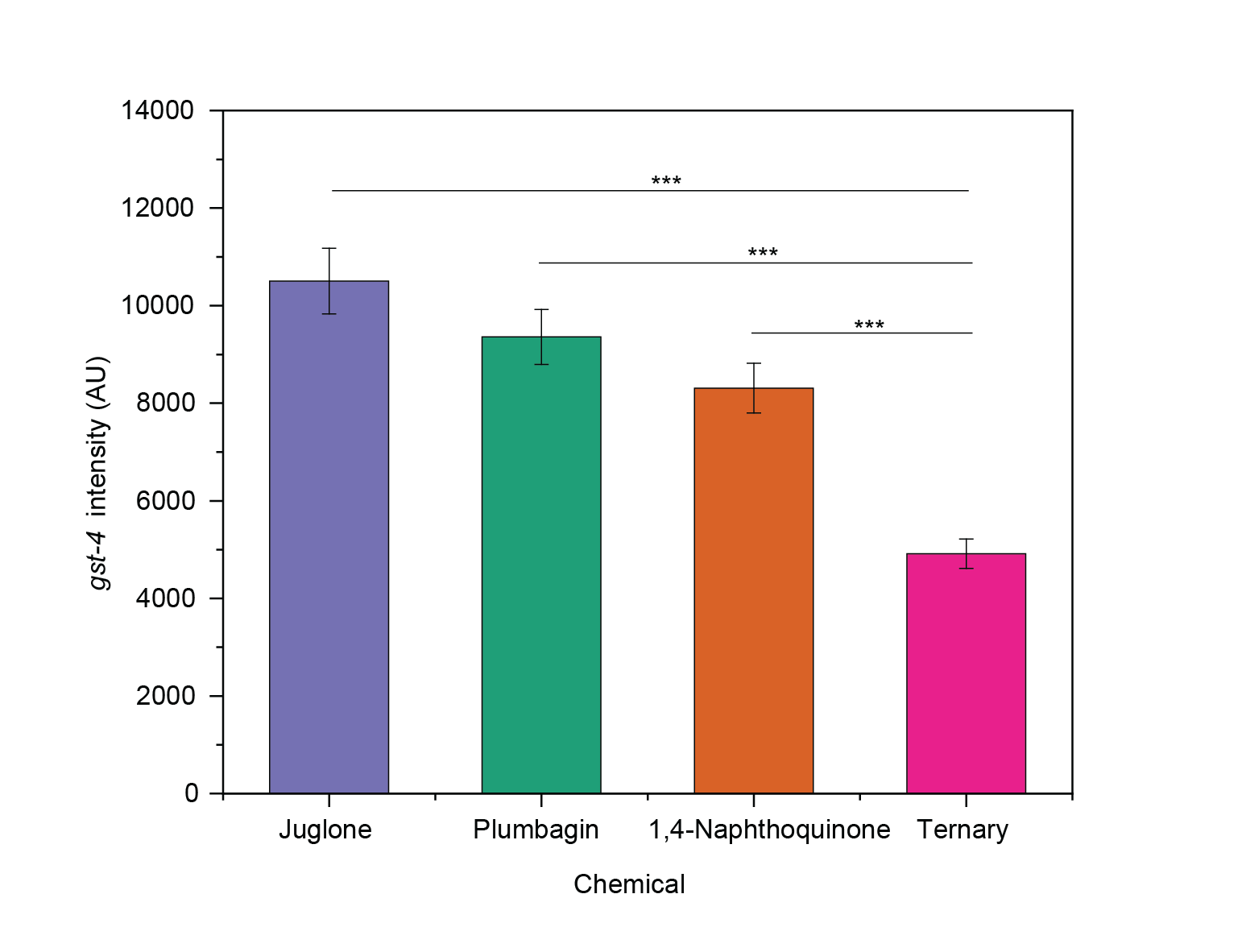


**Figure S2: *gst-4* response at 25 ºC.** *gst-4* response to Plumbagin, 1, 4-Naphthoquinone, Juglone, and ternary mixture at 25 ºC. p < 0.001 (***). Values follow the same trend as Figure 2B with a lower value for the ternary mixture (middle point of response surface in Figure 2B), and higher values for individual naphthoquinones (vertex points of response surface in 2B). Error bars are SEM.


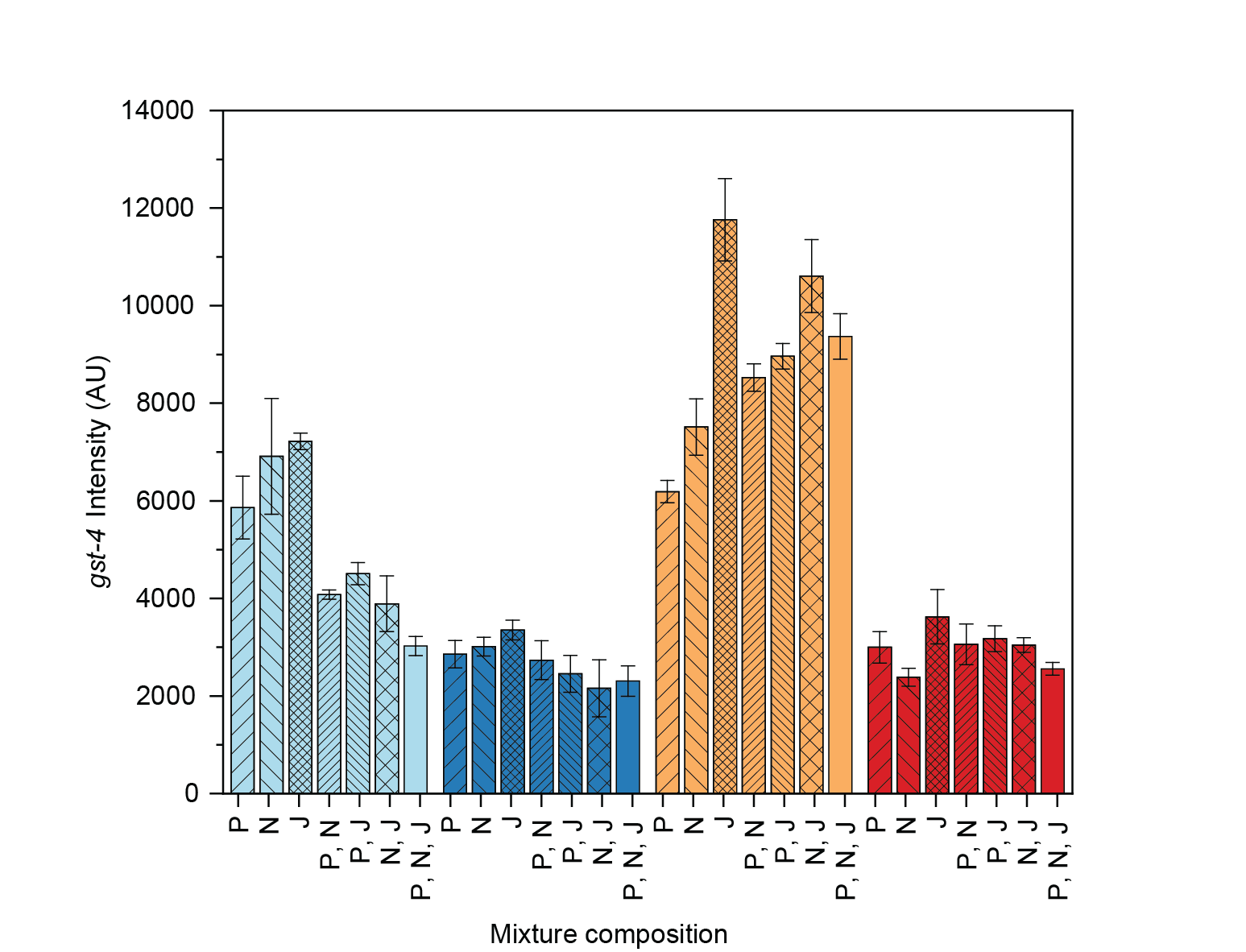


**Figure S3: Bar plot representation of experimentally acquired *gst-4* expression level.** *gst-4* expression levels of animals exposed to naphthoquinone mixtures at different process conditions. From left to right, AL 20ºC (light blue), AL 33ºC (blue), DR 20ºC (orange), DR 33ºC (red). P-Plumbagin, N-1,4-Naphthoquinone, J-Juglone. Error bars are SEM.


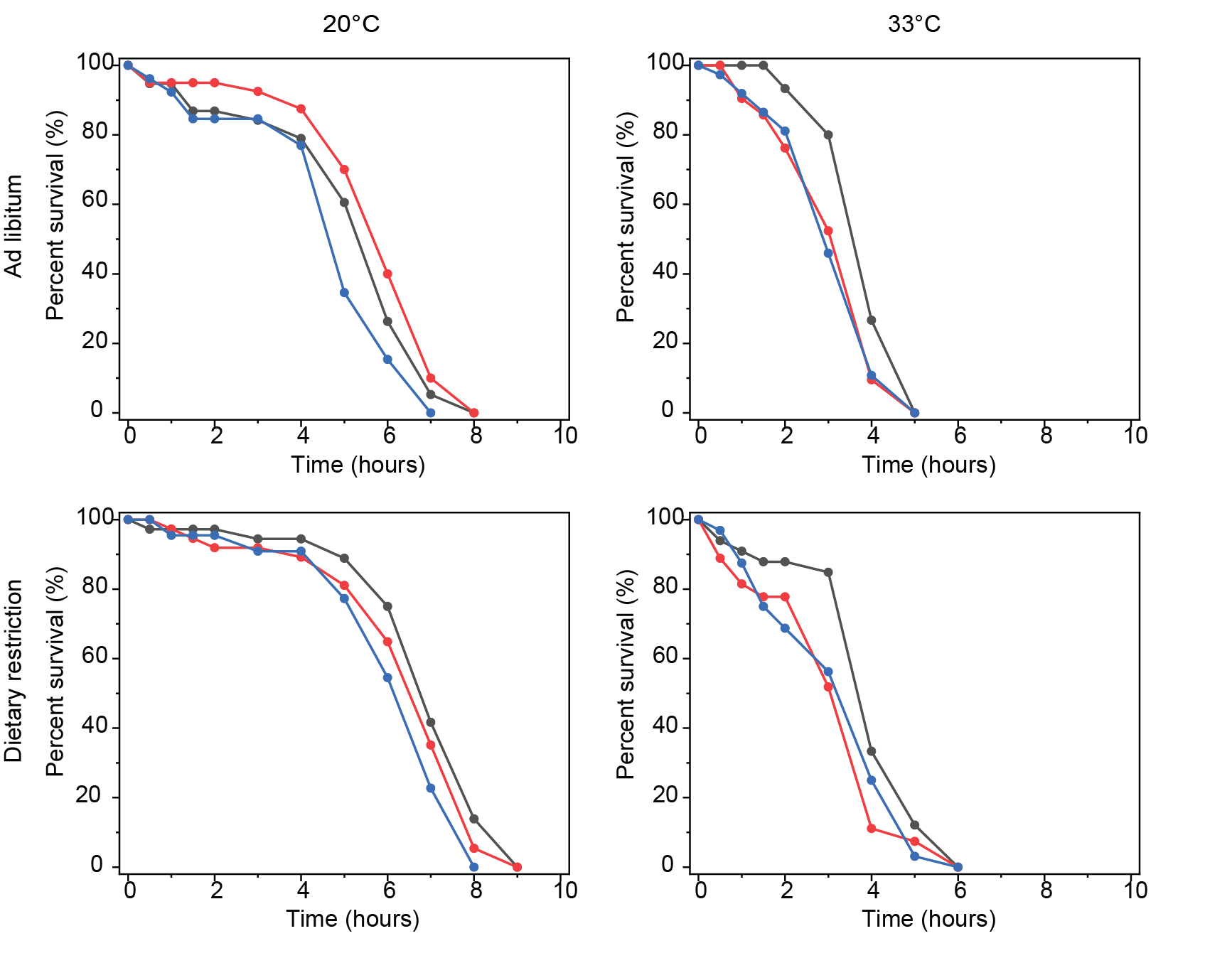


**Figure S4: Lifespan curves for juglone survival assay.** Lifespan curves built with OASIS 2 for CL2166 animals under ternary mixture at different environmental conditions. Each condition was tested three times.


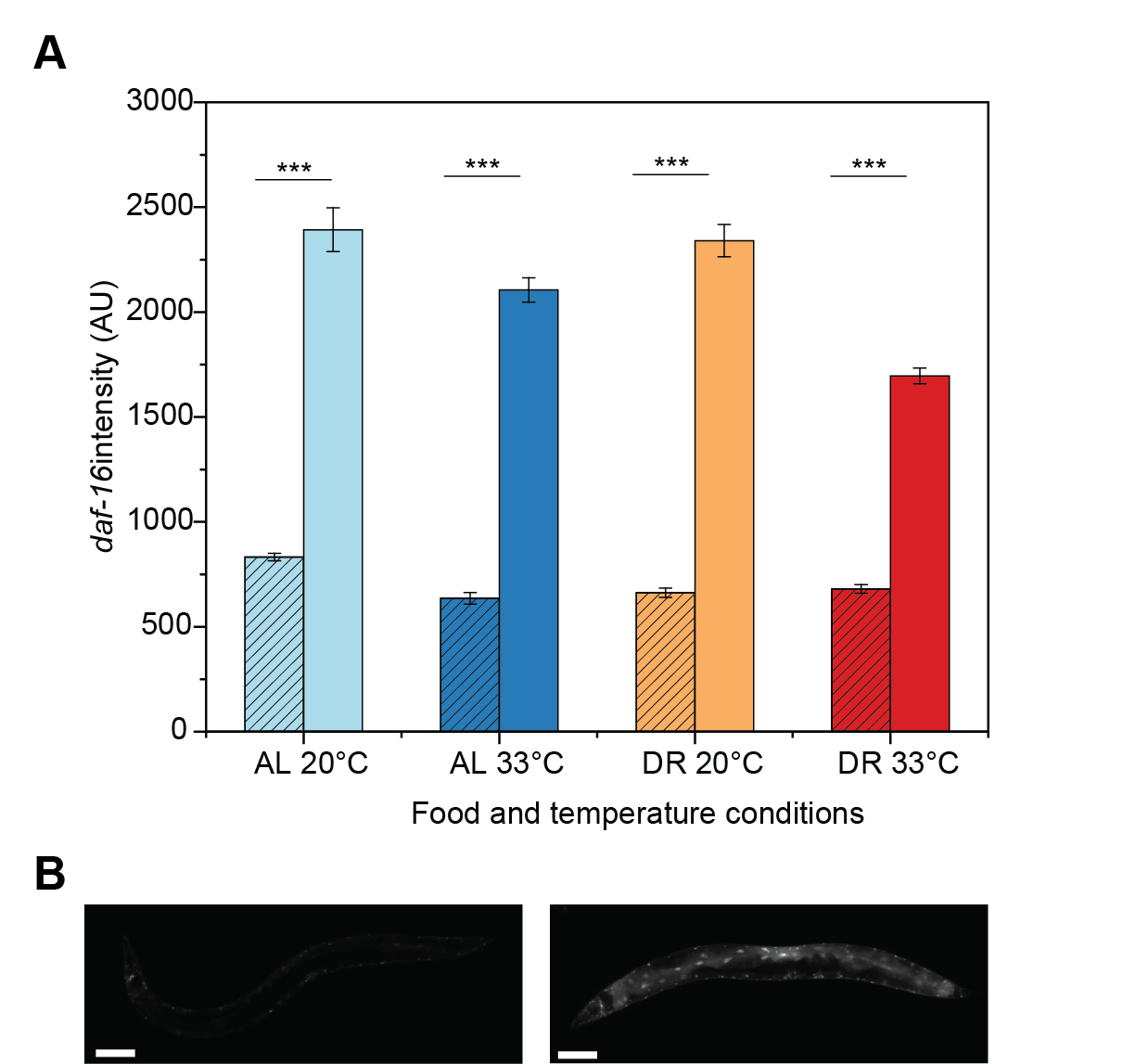


**Figure S5: *daf-16* response to ternary mixture.** A) *daf-16* response to ternary mixture at different environmental conditions. Striated bars represent MAH97 animals under *daf-16* RNAi, clear bars are control animals. B) Representative MAH97 animals under *daf-16* RNAi (left) and control (right). p < 0.001 (***). Scale bars are 100 µm. Error bars are SEM.

| **Table S1: Random effect prediction of population blocks and parameter estimates of main effects and interactions for naphthoquinone mixtures under ad libitum conditions.** | | | | | |
| --- | --- | --- | --- | --- | --- |
| *Term* | *BLUP/Estimate* | *Std Error* | *DFDen* | *t Ratio* | *Prob>\|t\|* |
| Population Block[1] | 275.37 | 321.85 | 20.79 | 0.86 | 0.402 |
| Population Block[2] | 300.83 | 322.62 | 20.75 | 0.93 | 0.3618 |
| Population Block[3] | -73.13 | 321.92 | 20.79 | -0.23 | 0.8225 |
| Population Block[4] | 724.31 | 322.62 | 20.75 | 2.25 | 0.0358 |
| Population Block[5] | 332.86 | 322.62 | 20.75 | 1.03 | 0.3141 |
| Population Block[6] | -1076.84 | 322.60 | 20.81 | -3.34 | 0.0031 |
| Population Block[7] | 161.88 | 321.13 | 20.77 | 0.50 | 0.6195 |
| Population Block[8] | 165.11 | 321.85 | 20.79 | 0.51 | 0.6133 |
| Population Block[9] | -119.89 | 321.43 | 20.75 | -0.37 | 0.7129 |
| Population Block[10] | -690.51 | 322.96 | 20.79 | -2.14 | 0.0446 |
| Juglone(Mixture) | 6647.05 | 339.80 | 29.88 | 19.56 | <.0001 |
| 1,4-Naphthoquinone(Mixture) | 5221.82 | 339.80 | 29.88 | 15.37 | <.0001 |
| Plumbagin(Mixture) | 4767.03 | 316.88 | 27.64 | 15.04 | <.0001 |
| Juglone*1,4-Naphthoquinone | -7270.80 | 1308.75 | 25.49 | -5.56 | <.0001 |
| Juglone*Plumbagin | -4855.49 | 1262.85 | 25.02 | -3.84 | 0.0007 |
| 1,4-Naphthoquinone*Plumbagin | -6065.19 | 1269.63 | 25.14 | -4.78 | <.0001 |
| Juglone*1,4-Naphthoquinone*Plumbagin | 10021.04 | 8789.28 | 25.07 | 1.14 | 0.265 |

| **Table S2: Random effect prediction of split plots and parameter estimates of main effects and interactions for naphthoquinone mixtures under simultaneous stress exposure.** | | | | | |
| --- | --- | --- | --- | --- | --- |
| *Term* | *BLUP/Estimate* | *Std Error* | *DFDen* | *t Ratio* | *Prob>\|t\|* |
| Whole Plots[1] | -575.96 | 450.16 | 14.27 | -1.28 | 0.2211 |
| Whole Plots[2] | 379.51 | 450.61 | 14.13 | 0.84 | 0.4137 |
| Whole Plots[3] | 310.30 | 450.16 | 14.27 | 0.69 | 0.5017 |
| Whole Plots[4] | 79.01 | 444.15 | 15.16 | 0.18 | 0.8612 |
| Whole Plots[5] | 986.22 | 445.56 | 15.25 | 2.21 | 0.0425 |
| Whole Plots[6] | 141.25 | 450.27 | 14.22 | 0.31 | 0.7583 |
| Whole Plots[7] | -26.32 | 446.61 | 14.85 | -0.06 | 0.9538 |
| Whole Plots[8] | 210.75 | 446.53 | 14.89 | 0.47 | 0.6438 |
| Whole Plots[9] | 79.75 | 444.79 | 15.02 | 0.18 | 0.8601 |
| Whole Plots[10] | -58.62 | 447.60 | 14.83 | -0.13 | 0.8976 |
| Whole Plots[11] | -290.92 | 450.17 | 14.24 | -0.65 | 0.5284 |
| Whole Plots[12] | -212.05 | 447.65 | 14.82 | -0.47 | 0.6426 |
| Whole Plots[13] | -433.91 | 446.53 | 14.89 | -0.97 | 0.3467 |
| Whole Plots[14] | -164.06 | 450.55 | 14.14 | -0.36 | 0.7212 |
| Whole Plots[15] | -147.08 | 453.01 | 13.66 | -0.32 | 0.7503 |
| Whole Plots[16] | 211.95 | 448.67 | 14.44 | 0.47 | 0.6437 |
| Whole Plots[17] | -57.95 | 476.77 | 12.05 | -0.12 | 0.9053 |
| Whole Plots[18] | 82.79 | 445.56 | 15.25 | 0.19 | 0.855 |
| Whole Plots[19] | -351.15 | 445.56 | 15.25 | -0.79 | 0.4427 |
| Whole Plots[20] | 296.32 | 446.92 | 14.86 | 0.66 | 0.5175 |
| Whole Plots[21] | -536.29 | 449.37 | 14.33 | -1.19 | 0.2521 |
| Whole Plots[22] | -645.85 | 447.61 | 14.83 | -1.44 | 0.1698 |
| Whole Plots[23] | -379.74 | 447.90 | 14.75 | -0.85 | 0.4101 |
| Whole Plots[24] | 360.88 | 448.96 | 14.40 | 0.80 | 0.4346 |
| Whole Plots[25] | 650.35 | 443.89 | 15.14 | 1.47 | 0.1633 |
| Whole Plots[26] | -450.20 | 447.77 | 14.81 | -1.01 | 0.3308 |
| Whole Plots[27] | -91.28 | 446.59 | 14.93 | -0.20 | 0.8408 |
| Whole Plots[28] | 632.30 | 453.01 | 13.66 | 1.40 | 0.185 |
| Juglone(Mixture) | 6447.75 | 295.31 | 55.91 | 21.83 | <.0001 |
| 1,4-Naphthoquinone(Mixture) | 5137.20 | 295.83 | 55.96 | 17.37 | <.0001 |
| Plumbagin(Mixture) | 4380.48 | 295.38 | 55.92 | 14.83 | <.0001 |
| Juglone*1,4-Naphthoquinone | -3024.96 | 1375.96 | 46.58 | -2.20 | 0.0329 |
| Juglone*Plumbagin | -2995.59 | 1343.95 | 42.43 | -2.23 | 0.0312 |
| Juglone*Dietary level | -1191.47 | 295.31 | 55.91 | -4.03 | 0.0002 |
| Juglone*Heat Stress level | -2927.53 | 295.31 | 55.91 | -9.91 | <.0001 |
| 1,4-Naphthoquinone*Plumbagin | 429.22 | 1350.27 | 42.74 | 0.32 | 0.7521 |
| 1,4-Naphthoquinone*Dietary level | -14.65 | 295.83 | 55.96 | -0.05 | 0.9607 |
| 1,4-Naphthoquinone*Heat Stress level | -2143.92 | 295.83 | 55.96 | -7.25 | <.0001 |
| Plumbagin*Dietary level | -392.97 | 295.38 | 55.92 | -1.33 | 0.1888 |
| Plumbagin*Heat Stress level | -1607.82 | 295.38 | 55.92 | -5.44 | <.0001 |
| Dietary level*Heat Stress level | 3198809.40 | 3503890.00 | 42.48 | 0.91 | 0.3664 |
| Juglone*1,4-Naphthoquinone*Plumbagin | -9811.49 | 9463.33 | 42.48 | -1.04 | 0.3057 |
| Juglone*1,4-Naphthoquinone*Dietary level | -4610.71 | 1375.96 | 46.58 | -3.35 | 0.0016 |
| Juglone*1,4-Naphthoquinone*Heat Stress level | 69.72 | 1375.96 | 46.58 | 0.05 | 0.9598 |
| Juglone*Plumbagin*Dietary level | -2178.32 | 1343.95 | 42.43 | -1.62 | 0.1125 |
| Juglone*Plumbagin*Heat Stress level | 1264.48 | 1343.95 | 42.43 | 0.94 | 0.3521 |
| Juglone*Dietary level*Heat Stress level | -3197723.00 | 3503871.00 | 42.48 | -0.91 | 0.3666 |
| 1,4-Naphthoquinone*Plumbagin*Dietary level | -2605.30 | 1350.27 | 42.74 | -1.93 | 0.0603 |
| 1,4-Naphthoquinone*Plumbagin*Heat Stress level | -507.70 | 1350.27 | 42.74 | -0.38 | 0.7088 |
| 1,4-Naphthoquinone*Dietary level*Heat Stress level | -3198449.00 | 3503874.00 | 42.48 | -0.91 | 0.3665 |
| Plumbagin*Dietary level*Heat Stress level | -3198617.00 | 3503869.00 | 42.48 | -0.91 | 0.3665 |
| Juglone*1,4-Naphthoquinone*Plumbagin*Dietary level | 3274.24 | 9463.33 | 42.48 | 0.35 | 0.7311 |
| Juglone*1,4-Naphthoquinone*Plumbagin*Heat Stress level | 7196.80 | 9463.33 | 42.48 | 0.76 | 0.4512 |
| Juglone*1,4-Naphthoquinone*Dietary level*Heat Stress level | 2508.55 | 1375.96 | 46.58 | 1.82 | 0.0747 |
| Juglone*Plumbagin*Dietary level*Heat Stress level | 1301.67 | 1343.95 | 42.43 | 0.97 | 0.3383 |
| 1,4-Naphthoquinone*Plumbagin*Dietary level*Heat Stress level | 1424.62 | 1350.27 | 42.74 | 1.06 | 0.2973 |
